# Hazardous Odor Recognition by CMAC Based Neural Networks

**DOI:** 10.3390/s90907308

**Published:** 2009-09-11

**Authors:** İhsan Ömür Bucak, Bekir Karlık

**Affiliations:** 1 Computer Engineering Department, Engineering Faculty, Fatih University, 34500, Istanbul, Turkey; 2 Computer Engineering Department, Engineering Faculty, Haliç University, 34381, Istanbul, Turkey; E-Mail: bekirkarlik@halic.edu.tr

**Keywords:** hazardous odors, electronic nose, CMAC neural networks, recognition

## Abstract

Electronic noses are being developed as systems for the automated detection and classification of odors, vapors, and gases. Artificial neural networks (ANNs) have been used to analyze complex data and to recognize patterns, and have shown promising results in recognition of volatile compounds and odors in electronic nose applications. When an ANN is combined with a sensor array, the number of detectable chemicals is generally greater than the number of unique sensor types. The odor sensing system should be extended to new areas since its standard style where the output pattern from multiple sensors with partially overlapped specificity is recognized by a neural network or multivariate analysis. This paper describes the design, implementation and performance evaluations of the application developed for hazardous odor recognition using Cerebellar Model Articulation Controller (CMAC) based neural networks.

## 1. Introduction

An electronic nose (e-nose) is an intelligent sensing device that uses an array of gas sensors of partial and overlapping selectivity, along with a pattern recognition component, to distinguish between both simple and complex odors. To date, e-noses have had a variety of use in a number of applications such as the food industry, medical diagnosis, mobile robots, environmental disasters or intelligent appliances [1–10].

The monitoring of the quality of air in an enclosed environment has always been an important concern. Hazardous odor (or gases) can be present as a result of leaks in tanks, piping, etc., and their presence needs to be monitored to prevent the accidental exposure to a hazardous condition. Analytical chemistry instruments such as gas chromatographs (GC) and mass spectrometers (MS) have been used to analyze both hazardous and non-hazardous odors. GC and GC/MS systems can require a significant amount of human intervention to perform the analysis and then relate the analysis to something usable [4–5]. The odor sensing system should be extended to new areas since its standard style where the output pattern from multiple sensors with partially overlapped specificity is recognized by a neural network. In the last decades, the use of environmental monitoring has been rediscovered due to major advances in odor sensing technology and soft computing techniques such as artificial neural networks (ANN), fuzzy systems and the other artificial intelligence techniques [6–10].

Nonlinear response characteristics and the use of an array of gas sensors have made artificial neural networks very attractive because of their capability to analyze multidimensional nonlinear sensor data, and to model sensor response, which is mathematically very difficult. In the past, work has been done on chemical gas sensors using Multilayer Perceptron (MLP) artificial neural networks. Gas sensor calibration is one of them [11]. However MLPs present a major disadvantage of slow training. This drawback makes them unsuitable for real time training and adaptive modeling. They require much iteration to converge and a large number of computations per iteration.

In this paper, a fully operational CMAC based neural network recognition system which models the function of the biological nose is presented and applied to recognize hazardous odors. One of the main advantages of CMAC based neural networks compared to MLP is their extremely fast learning capability. Different from MLPs, CMACs have simpler calculations, higher convergence speed and better generalization ability, and non-existing local minima. Therefore, they are widely applied in controls and real-time recognition problems [12–13].

The remaining of the paper is arranged as follows. Section 2 briefly explains the basics of the CMAC neural network and its significant properties. Section 3 describes odor recognition using the CMAC neural network including training mode, test mode, and presents the steps of the recognition algorithm. Section 3 explains the MLP algorithm briefly, as well. Section 4 discusses the simulation results including comparisons between the two algorithms. Finally, conclusions are drawn in Section 5.

## 2. Cerebellar Model Articulation Controller (CMAC) Neural Networks

An artificial neural network is used in the recognition and classification of different odors and is constructed as a standard multilayer feed-forward network trained with the back-propagation or the other combined neural networks algorithms [6–10].

**Figure 1.**
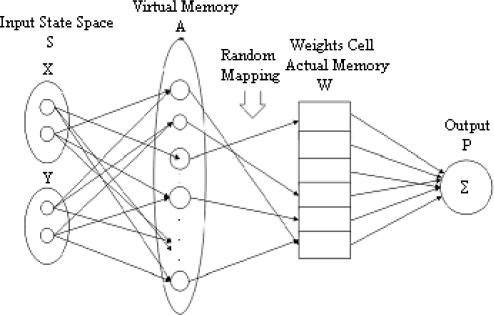
A block diagram of a CMAC.

The CMAC was firstly proposed during the 1970s by James Albus, whose idea was based on a model of the cerebellum which is a part of the brain responsible for learning process [14]. The CMAC can generally be described as a transformation device that transforms given input vectors into associated output vectors [15]. The CMAC is an algorithm that quantizes and generalizes input, produces active memory addresses, and produces an output by summing all the weights in the active memory addresses [16]. This process of finding the output has several steps. Figure 1 shows a CMAC functional diagram that has two inputs and one output.

**Figure 2.**
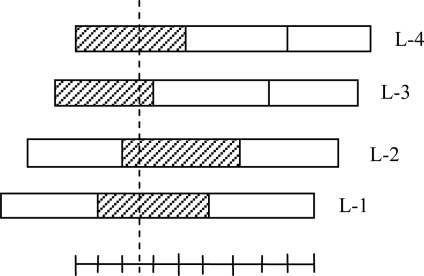
Receptive field organization.

In a two-input typical CMAC, each variable in the input state vector is fed to a series of input sensors with overlapping receptive fields. The total collection of receptive fields is divided into *C* subsets, referred to as *layers,* which represent parallel *N*-dimensional hyperspaces for a network with *N* inputs. The receptive fields in each of the layers have rectangular boundaries and are organized so as to span the input space without overlap. Figure 2 shows an organization of CMAC neural network receptive fields for a one dimensional case [17].

Any input vector excites one receptive field from each layer, for a total of *C* excited receptive fields for any input. In Figure 2, the total number of excited receptive fields is 4 (i.e., *C* = 4) where the hatched regions show the active or excited fields. Each of the layers of receptive fields is identical in organization, but each layer is offset relative to the others in the input hyperspace. The width of the receptive field of each sensor produces input generalization, while the offset of the adjacent fields produces input quantization. Each input variable excites exactly *C* input sensors, where *C* is the ratio of generalization width to quantization width. Each input sensor produces a binary output which is ON if the input falls within its receptive field and is OFF otherwise. The binary outputs of the input sensors are combined in a series of threshold logic units (called state-space detectors) with thresholds adjusted to produce logical AND functions (the output is ON only if all inputs are ON). Each of these units receives one input from the group of sensors for each input variable, and thus its input receptive field is the interior of a hypercube in the input hyperspace [18].

The leftmost step in Figure 1 presents the physical input state space. It may contain one or more input vectors (Figure 1 shows two). These vectors are composed of discrete points. These discrete points are connected to the second step of the CMAC known as state space detectors. The state space detectors are often called the CMAC’s virtual memory. This transformation contains quantization process and input generalization with generalization factor (width) [15]. Input sensors overlap and cover *width* number of inputs. Therefore, *width* is used to indicate the number of inputs covered by overlapped input sensors. Input values are quantized into one of *quant* values and hence *width* can vary between 1 to *quant*. Low numbers usually work best [16].

A vector of quantized input values specifies a discrete state and is used to generate addresses for retrieving information from memory for this state. Each state variable is quantized into discrete regions, called *blocks*. It is noted that the width of blocks affects the generalization capability of the CMAC. The number of blocks in CMAC is usually greater than two. The output generalization capability of CMAC is controlled mainly by the width of the blocks. If two inputs are far apart in the input space, there will be no overlap and as the result, no generalization [19].

Quantization has been used due to the fact that the minimum variations in the input values do not affect the output values. Quantization levels affect the values of the input vector. The stability of inputs depends on the level of quantization. If the quantization level increases, the stability of inputs increases.

**Figure 3.**
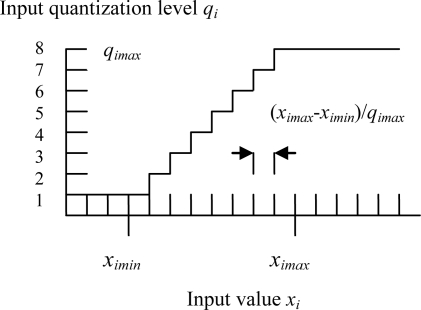
CMAC input quantization.

The resolution of the quantization depends on the expected maximum and minimum input values (See Figure 3 for input quantization) [20]. The quantization and mapping between input space and virtual memory give the CMAC the ability to the input generalization which means that the CMAC has the property that any two input vectors that are similar or close in the input space will select a highly overlapping subset of locations in the state space during mapping between input state and state space detectors. Thus, the output response of the CMAC to similar input vectors will tend to be similar because of many memory locations that are in common. Hence, the CMAC tends to local generalization. The amount of generalization depends on the number of overlapping memory locations in the state space detectors.

The next step is mapping from the state space detectors into the physical memory. This mapping process may be realized in two different ways. First one is one-to-one mapping and the other one is many-to-one mapping or random mapping. Since the physical memory is assumed to be smaller than the number of state space detectors, this random mapping is a many-to-one mapping, and sometimes causes collisions [15]. The general rule of thumb to map from virtual memory to physical memory indicates that if the state space detectors are not small enough for one-to-one mapping with physical memory, then random mapping should be used. In other words, if the state space detectors are small enough for one-to-one mapping with physical memory, we should use one-to-one mapping. The last step includes summing all the weights in the physical memory to produce the output vectors. During training, if the output vectors of the CMAC do not match a desired output for a given input state, the weights pointed to by the physical addresses are updated using the following least mean square (LMS) rule (also called the delta rule), which results in the steepest descent to the error minimum (a.k.a. steepest descent update rule) [20]:

(1) $$w_{j_{\left(\mathrm{new}\right)}}\leftarrow w_{j_{\left(\mathrm{old}\right)}}+\beta \frac{\left(y_{d}-y\right)}{g}$$

This is a supervised learning equation whose convergence can be guaranteed. The objective is to find the weights which minimize the error defined as the difference between desired and realized outputs. In Equation (1), *w_j_* is the weight, *y_d_* is the desired output of the CMAC system, *y* is the actual output of system, *g* is the number of associated memory cells (a.k.a. amount of network generalization), and *β* is the learning rate or the learning coefficient.

For a specific input quantization mapping, an increase in *g* means an increase in the amount of shared weights between neighboring input/output pairs. An increase in the number of quantization levels, *q_imax_*, results in higher input resolution (Figure 3), but concurrently increases the size of the virtual address space, and hence slowing speed [21–22].

Constant learning rate *β* scaled between *0* < *β ≤ 1* can produce unstable learning behavior in certain situations if the value of *β* is too close to 1. Specifically, the learning speed will improve for a large *β* but there will be error due to gradient noise. A smaller learning rate will result in smaller adjustments to the look up table and thus slow training.

The CMAC has several potential advantages over other neural network structures. Specifically the CMAC neural network has been preferred over the multilayer neural network; because the multilayer network requires many iterations and a large number of computations per iteration to converge an output so that the algorithm runs slowly [23]. However, the CMAC presents many attractive features and advantages, and is useful for real time applications. The CMAC has been used to solve various robotic problems, and applied in the field of controls, medical science, pattern recognition, signal processing and image processing [23,24].

## 3. Odor Recognition using CMAC Neural Network

In this study, an OMX-GR odor meter, manufactured by Shin-ei Co., is used to obtain odor data or measure ambient odor. This meter is a “hand-held” odor monitor and can measure relative values of odor. A metal-oxide semiconductor gas or odor sensor, i.e., OMX-GR, is engaged as an odor detecting element. Sensitivities of OMX-GR sensors are explained by two factors, strength and classification [9]. This provides a lot of benefits for such applications regarding odor detection and measurement. This sensor can detect various odors and sweet smell of gases or reducing gases as measuring objects or substances, however it cannot detect oxidizing gas like ozone. In one of the applications, this odor sensor has been used to prevent *Lilium auratum* (a perennial herb) flowers of sampled individuals from being pollinated by using gas chromatography and this OMX-GR sensor [25]. Morinaga *et al.* in [25] used this sensor because of its convenience in collecting many data in the field, which is required for odor sensor analysis at some certain sampling period.

**Figure 4.**
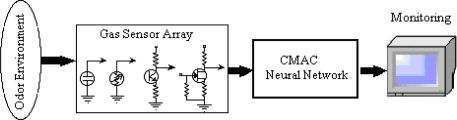
Odor recognition system.

In this study, a high performance odor recognition system with the capability of discriminating four different hazardous odor patterns, which are CO, acetone, ammonia, and lighter gas, is developed; hence a real-time classification method using a hand-held odor meter (OMX-GR sensor) and biologically inspired CMAC based artificial neural network are proposed. The system allows users to obtain the desired hazardous odor data through either real-time sampling or memory sampling using the sensor and to analyze the data by using the proposed CMAC neural network algorithm as a pattern classifier. The OMX-GR odor sensor acquires the data through a PC to use the CMAC neural network classifier as input. The schematic diagram of the entire system is illustrated in Figure 4 below.

The multiple OMX-GR odor sensor signals are simultaneously measured using strength of odor concentration. The system consists of two semiconductor gas sensors (OMX-GR). These two sensors operate in a real-time sampling mode, which is a continuous sampling with a built-in air pump. The system is based on different sampling intervals. Those variable sampling intervals are 1, 2, 5, 10, 20, 60, 120 or 300 seconds. 26,176 data points can be stored. This capacity can be partitioned into 16 files (one file per 1,636 data points) [26]. An equally sized four groups of data samples, i.e., 256 (64 × 4) were stored in seconds for the hazardous odors. Seventy percent of these data were used as for training and the other thirty percent for testing. Different sampling interval can be allocated into the file. Its operation temperature is between 0 and 40 degrees Centigrade. Real-time continuous data can also be stored into a personal computer through RS-232C interface. Then, the multiplexed time-series data, which belongs to four different hazardous odors, are inputted to the CMAC neural network algorithm which is trained to classify these hazardous odors.

Table 1 shows the results of the CMAC neural network algorithm for the odor data for various learning rates while quantization and width parameters are kept unchanged. As the learning rate *β* is increased, it takes shorter steps for the algorithm to classify the input states accurately; this comparison among various learning rates can be seen well as the desired errors of each specifically designated learning rate value get smaller. However, this result comes with an additional cost of the initialization time which grows up slightly, therefore resulting in a bit of sacrifice in the learning speed. Number of learning steps (*k*, an integer) means that the algorithm learned to classify the data successfully at *k* iterations or pass as determined by the desired error.

Each data set has been normalized according to Equation (2):

(2) Normalized_value=(current_value−(min_value−1))/((max_value−min_value)+2)

According to Equation (2), the entire range of the odor data is normalized to vary between 0 and 1, and thereafter the normalized data is used to train and test the CMAC artificial neural network.

### 3.1. Training Mode

In the training mode, the normalized odor data are used to train the CMAC ANN. These data perform the mapping process first between quantization and memory locations to start with the network training after being loaded into the CMAC ANN. Later the output vector is formed by summing the weights in the physical addresses so that the training process gets done. The recognition is decided upon the similarity process which seeks similarity between the output vector of the test data and the training data after the test data has been gone through the similar process as the training data.

Training is essentially the weight update process in which the actual output with the desired output is compared, and then the weights of the active neurons, if a difference exists between the actual and desired outputs, are updated with the predetermined learning rate according to the LMS rule (Equation 1). This is basically no different from updating the weights of the active neurons.

### 3.2. Test Mode

The CMAC ANN should be able to classify correctly the input vectors which were never seen before. For this reason, the totally different data from the data of the training, which goes through the same normalization process and is called test data, is input to the network for the recognition process. The operations of the CMAC ANN will be the same as the training mode when the test data is inputted to the recognition system. In this mode, the weights of the same excited memory addresses of each memory layer are summed up and each layer has one output value. If the input signals are the same as the training patterns, they will excite the same memory addresses [22]. Hence, the output of CMAC ANN can be any one of the hazardous odor types, CO, acetone, ammonia, or lighter gas.

### 3.3. The Algorithm

The CMAC algorithm is described as follows:

Step 1: Build configuration of the CMAC odor recognition system.
Step 2: Normalize, load and input the *training* data, through quantization, memory addressing, and the weights of the summation of excited memory addresses to produce the output nodes.
Step 3: Calculate the difference between actual output and desired output to find the weights, which minimize the error as based on the LMS rule [Equation (1)].
Step 4: Training is done! Save the memory weights.
Step 5: Normalize, load and input the *testing* data, through quantization, memory addressing, and the weights of the summation of excited memory addresses to produce the output nodes. (If the input signals are the same as the training patterns, they will excite the same memory addresses.)
Step 6: Output the testing result.

### 3.4. The Algorithm of MLP

The most common neural network model is the multi layered perceptron. An MLP network is grouped in layers of neurons, that is, input layer, output layer, and hidden layers of neurons that can be seen as groups of parallel processing units. Each neuron of a layer is full connected to all the neurons of the following layer (feed-forward neural network). These connections are directed (from the input to the output layer) and have weights assigned to them [9]. For comparing with CMAC, a MLP trained with the back-propagation algorithm was used for recognition of collecting data of hazardous odors.

**Figure 5.**
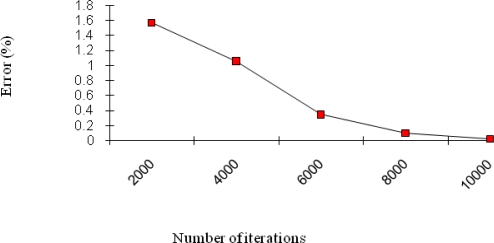
Total MSE for number of iterations in the MLP.

The MLP structure of this study was 8:8:4 which mean the number of neurons in each layer. The optimum learning rate (*β*) was found 0.7 by a trial-and-error method. Figure 5 shows a total Mean Square Error (MSE) for the different number of iterations in the MLP structure. If we increase the iteration number of the neural network, the error gets decremented as can be seen from the figure. But overall, this architecture requires a very large number of iterations as compared to that of the CMAC.

## 4. Simulation Results

**Figure 6.**
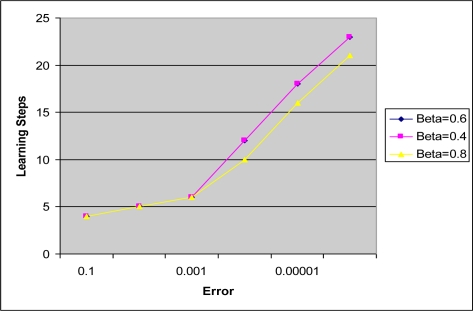
Learning step vs. desired error for various learning constants, *β* (for quant = 4 and width = 2).

Figure 6 shows that, as the learning rate *β* is decreased, it takes longer steps for the algorithm to classify the input states accurately. This is another way of expressing the results of Table 1, and may be used as a better tool to describe the results through a visual comparison. The learning rate 0.8 may be chosen to work the best of all the three considered in the figure. Please note that the 0.6 and 0.4 learning curves overlap and therefore follow almost the same path in the figure.

**Figure 7.**
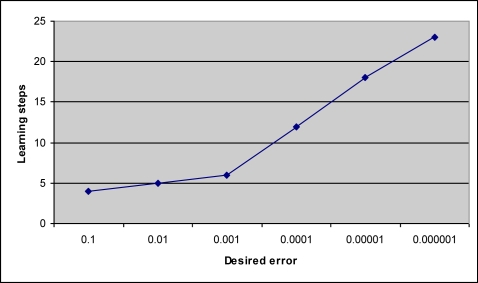
Learning step vs. desired error for quant = 4 and *β* = 0.4.

Figure 7 indicates that any desired error up to 0.001 will make almost no difference for the learning steps which means approximately the same amount of learning time for the algorithm to learn to classify correctly. That can also be seen from the resulting data in Table 1. However, as the desired error is reduced more, the steps for the algorithm to learn to classify the data correctly will increase linearly in this particular problem, i.e., taking more steps or time than before to learn.

**Figure 8.**
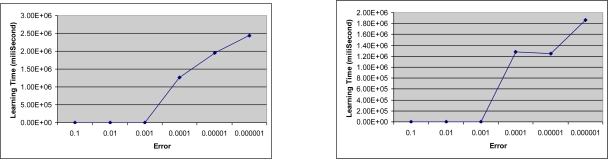
Learning times of the desired errors (a) for quant = 4 and *β* = 0.6 (b) for quant = 4 and *β* = 0.4.

Figure 8 (a) and (b) show the relationship between the desired error and the learning time when the input quantization is 4 for both, and the learning rates are 0.6 and 0.4 respectively. In both figures, there is no any recordable variation in the learning time until the desired error becomes 0.0001. Nonetheless, the learning time increases sharply as the desired error becomes smaller than 0.0001 as seen in the figures.

The input data was presented to the neural networks without weight adjustment for testing. The average recognition rates were found 85%, 99%, 100, and 99% respectively for each gas, as shown in Table 2.

The total MSE error was found 0.0235% after 10,000 iterations using ordinary MLP architecture [26]. We observed that the error was reached on CMAC accuracy level after 10,000 iterations.

## 5. Conclusions

The electronic nose developed in this research consists of a sensor array in which each sensor gives a different electrical response for a particular target vapor introduced into the sensing chamber. Pattern recognition techniques based on the principal component analysis and the CMAC neural network model have been developed for learning different chemical odor vapors. This study has demonstrated the feasibility of an electronic nose and the CMAC neural network to detect and identify some of the hazardous odors. Hundred percent success rate of classification was accomplished with the design of CMAC ANN architecture for hazardous odor recognition system.

The other well-known ordinary MLP architecture is also able to generalize with very high recognition accuracy. However, the training time of MLP is longer than CMAC. In the near future, some other neural network based classifiers and Bayesian classifier are planned to be used for comparison purposes to recognize hazardous odors.

## Figures and Tables

**Table 1.** Results of the CMAC Neural Network Algorithm for the odor data.

**Q**	**W**	**β**	**Desired Error**	**Init Time (s)**	**Learning Time (s)**	**Test Time (s)**	**Step**
4	2	0.4	0.1	1,390	0.438	0.031	4
4	2	0.4	0.01	1,375	0.578	0.031	5
4	2	0.4	0.001	1,391	0.640	0.031	6
4	2	0.4	0.0001	1,359	1,282	0.015	12
4	2	0.4	0.00001	1,344	1,250	0.032	18
4	2	0.4	0.000001	1,375	1,860	0.031	23

4	2	0.6	0.1	1,359	0.453	0.047	4
4	2	0.6	0.01	1,375	0.562	0.016	5
4	2	0.6	0.001	1,391	0.640	0.031	6
4	2	0.6	0.0001	1,390	1,266	0.015	12
4	2	0.6	0.00001	1,391	1,953	0.016	18
4	2	0.6	0.000001	1,391	2,438	0.015	23

4	2	0.8	0.1	1,391	0.469	0.031	4
4	2	0.8	0.01	1,375	0.578	0.016	5
4	2	0.8	0.001	1,390	0.657	0.015	6
4	2	0.8	0.0001	1,360	1,094	0.031	10
4	2	0.8	0.00001	1,407	1,656	0.031	16
4	2	0.8	0.000001	1,406	2,250	0.032	21

**Table 2.** The recognition results for testing of neural networks.

**Type of Gas**	**Recognition rates for Validation (%)**	**Recognition rate for Test (%)**
CO	97	85
Acetone	98	99
Ammonia	99	100
Lighter	98,5	99
